# Social accountability: a survey of perceptions and evidence of its expression at a Sub Saharan African university

**DOI:** 10.1186/1472-6920-12-96

**Published:** 2012-10-18

**Authors:** Moses Galukande, Noeline Nakasujja, Nelson K Sewankambo

**Affiliations:** 1 College of Health Sciences, Makerere University, Kampala, Uganda; 2 Department of Surgery, College of Health Sciences, Makerere University, P.O. Box 7072, Mulago Hill Road, Kampala, Uganda

**Keywords:** Social accountability, Expression, Perception

## Abstract

**Background:**

Many medical schools express a commitment to social accountability. However there are significant short comings in the ways doctors are educated with respect to the social contract between medicine and society. Being socially accountable in the context of a medical school is to conduct health, research and training activities in such a way that best prioritises the health needs of the people served. However, there is little clarity among medical educators on what is meant to be socially accountable.

**Methods:**

This study sought the perceptions of senior medical educators and students on the concept and evidence of expression of social accountability in at Makerere College of Health Sciences through a cross sectional descriptive qualitative study. Twelve key informative interviews were conducted. The recorded interviews were transcribed and findings analyzed through a collaborative thematic approach.

**Results:**

Social accountability was not a familiar concept and had not been encountered by many of the key informants. However, the respondents contented that it is the individual’s responsibility to be ‘sensitive’ to the needs of the communities the individual serves. The respondents made it apparent that the schools’ emphasis on community based training and service among other efforts demonstrate social accountability. There were challenges though that impeded strengthening this position, like the lack of resources in the community to support continued students and faculty stay and a lack of resources to conduct translational research activities from a pre determined research agenda.

**Conclusions:**

Despite a general unfamiliarity of the concept, there was compelling evidence in way of substantial effort and measurable outcomes, that this school has been socially accountable for a long time. However, there is need for increased awareness and a deliberate strategy to improve social accountability in a resource limited context by articulating a model to guide further implementation of the College’s intentions.

## Background

Many medical schools express a commitment to social accountability however the task of evaluating and assessing the extent to which medical schools are socially accountable is complex [1]. The complexity arises from the fact that the judgment of how well a school is doing depends a lot on the context (social, political and geographical) in which it operates. The commitment to social accountability has resulted from an observation that there are significant shortcomings in the way doctors are educated with respect to the social contract between medicine and society. Social accountability by medical schools is defined as “an obligation to direct education, research and service activities towards addressing the priority health concerns of the community, region, and/or nation they have a mandate to serve [2-5]. It is suggested that there are four principles that should be expressed in social accountability activities: relevance, quality, cost effectiveness and impact. Evidence of the principles is looked for during quality assurance audits and is embedded in accreditation standards for medical education. Whereas accreditation standards of medical education are in practice in many parts of the world where they are of importance in ensuring quality education and should reflect social accountability, little is known about them in the African setting, [6-8]. There is little clarity among medical educators on what it means to be socially accountable [2]. Neither do we know how social accountability is best taught or learned. Little is known about the forms of social accountability, effective teaching and evaluation or the preparedness of future physicians to be socially and community responsive. Given the fact that Makerere Medical School now called the Makerere College of Health Sciences (MakCHS) for several decades was the only medical training institution in the region by default, it served the purpose of providing health workers especially doctors who served competently in many of the facilities in the region and beyond yet no documentation on social accountability exists. With this background we set out to determine the perceptions on the understanding, teaching and learning of social accountability within one of the oldest medical schools in the Sub Saharan African.

## Methods

This was a cross sectional qualitative study conducted in 2008 at MakCHS, Uganda. A rapid inexpensive self evaluation approach was employed. Twelve face to face key informant interviews were conducted including senior medical educators and two student leaders. Face to face interviews were adopted as a method of data collection given its rapid nature of gathering information from a community of interest. The research by interview method was employed as it allowed for seeking of perceptions and impressions of the respondents while allowing for the opportunity to probe and ask follow up questions. The key informants were deemed well positioned to provide detailed information and opinions based on their knowledge, experience and position.

For a long time, its (College of Health Sciences) main role was to train medical doctors but over the years other forms of health professionals have also graduated from this institution; including nurses, dentists, pharmacists, public and environment health graduates.

Over several decades MakCHS was the only training institution for doctors in the East African region and indeed its graduates populated most of the hospitals and Ministries of Health as leaders in health care provision. Without having defined social accountability, the way we now know, it was being practiced any way. Close to a decade now, the MakCHS changed the teaching and learning methods from the old teacher centered curriculum to problem based learning (PBL). With students going out to the communities for service learning through community based education and service (COBES) activities, this was seen as one of many moves towards a socially accountable institution.

The sampling of the key informants was purposive; selecting deans and heads of different units with at least 15 years of teaching and managerial experience. Those in leadership and with a long experience in academia at the institution were deemed most appropriate to provide the type of knowledge and practice of social accountability sought after in this particular study. Involvement of students’ representatives was justified as representation of students’ body. A list of the 24 eligible participants was generated and each called on phone to ascertain availability and willingness to participate. Eighteen were reached on phone and were available and willing to participate. An interview schedule was made, 12 honored the interview appointments, 4 had other commitments, and 2 failed to attend the scheduled appointment. The entire exercise (the Interview schedule) lasted 3 weeks.

The information was collected by N.N and M.G through audio recorded interviews with permission from the respondents. Each interview lasted 40–45 minutes. A general interview guide approach was employed because it ensured that the same general areas of information were collected from each interviewee; this provided more focus than would have been the case if we used an informal conversational approach, but it also allowed a degree of freedom and adaptability in this information gathering process. The information was transcribed verbatim using Microsoft word processing program. Textual data was structured in matrices with rows for the thematic areas, this allowed for comparison of statements among the respondents. The transcripts were reviewed repeatedly through a collaborative analytic process to generate codes and emerging themes.

The study received ethical approval from the University College based Research and Ethics Committee.

## Results

### Definition of the term

There were ten educators and two student leaders interviewed. The term social accountability was not a familiar term to the key informants. Half of them had not previously encountered the term and it was vague to the rest of the respondents as exemplified by this respondent:

"“Yes I have but not very frequently”."

The term was clarified to interviewees after collecting their views.

The concept of social accountability within medical practice and education was thought to be a duty of individuals towards a community by providing service and training medical students to be more sensitive to the community needs that they serve as expressed in this quote:

"“It borders with social responsibility….one has to be responsible for health materials in order that that they should not be embezzled or wasted. Consumers should be able to question how I use the resources that are meant for them.”"

This statement could be taken for ensuring equity of service provision. Emphasis is laid on the point that corruption impedes access to quality services.

### Expression of social accountability in the college

The training of health workers was looked at as a major contribution to social accountability [9]. The PBL method [10] of teaching and learning was viewed as a means through which social accountability is realized as students learnt through real life problems and hopefully mastered the skills of problem solving thereby becoming lifelong learners. It would then translate into solving community priority problems. The COBES program places students of each class once a year in a rural health care facility. The activities carried out involve providing a service to those that need it. This is now known to improve quality of service delivery in the communities where the students go. It is also known to increase the chances of students once they graduate, to work in underserved areas [11,12]. Exposure to real life rural situations endears many students to these communities as shown in the statements made by the students’ respondents below:

"“COBES helps future health workers to be exposed to the community they are meant to serve upon graduation. Stakeholders are invited to participate in curriculum development in order to improve relevance of training to community needs…”"

Whereas training was believed to be an act of social accountability, it is an assumption that is usually wrong [13]. The trained workers have to be seen getting involved in solving community priority health needs.

Deployment of the students to the community sites for COBES curriculum activities and by the school staff offering services at the national referral and teaching hospital was seen as evidence of social accountability and here are quotes in that regard;

"“There is training of health care providers by the College. Research in carried out in the community….COBES offers service to the community……”"

"“….Students give back to the community through the projects done in the community…. they volunteer at Internally Displaced Peoples’ camps and, do research around Mulago hospital….”"

These assumptions may only be right if there is evidence that priority health needs are met. However it was also felt that sometimes the service was both direct and indirect as stated by one of the respondents:

"“Service to the community is done directly and indirectly by staff helping out in provision of health care, teaching at the hospital…….”"

Furthermore, the current expression of social accountability was articulated by the Head of the College as shown in Table 1 below.

**Table 1:** **The current expression of social accountability at the CHS**^**#**^

**Domain**	**Expression**
Mission	Mission is explicitly oriented to social accountability **
Functional status change	Change from Faculty of Medicine to College of Health sciences incorporating 4 schools (School of: Medicine, Biomedical Sciences, Health sciences and Public Health) enhancing inter schools partnerships to enhance a holistic approach to addressing health challenges.
Curriculum reforms	In the past decade we strengthened community outreach activities by adopting the COBES† concept as part of curriculum implementation strategy. COBES enhances the local relevance of our education to community needs and quality of services delivered to the communities.
Impact of curriculum revision	Increase in OPD attendance has been registered at over 40 rural Health Service delivery sites, over the past several years as well.
	Increased motivation of workers.
Curriculum Review	Currently a curriculum review is underway, it’s been preceded by a needs assessment exercise intended to integrate and align the Ministry of Health agenda with college’s strategic plan and subsequent curriculum activities.
Accreditation	We undertook to develop standards of Medical Education, building on WFME* work. Standards positively reflect social accountability intent and practice.
Professionalism	A longitudinal study was started a year ago, to better understand how to inculcate professionalism better in trainees as a positive way of enhancing social accountability. Lack of professionalism under mines access to health care.
Community concerns	Research, and outreach services to the neighbor ring slums was contacted over a 3 year period, included mass deworming and immunization along with health talks about sanitation among other activities.
Partnerships	A Medical Education Partnership Initiative (MEPI) grant has been secured for a national wide effort to address Quality of Education Training and Community Research and Service.

CHS- College of Health Sciences, Makerere University.

† COBES – Community Based Education and Service.

* WFME – World Federation of Medical Education.

** Old mission - “we are dedicated to improving the health of the people of Uganda and beyond and promoting health equity by providing quality education, research and health services. We achieve this by enhancing capacity and participation of stakeholders; strengthening systems and partnerships; and harnessing the power of new sciences and technology so as to build and sustain excellence and relevance.”

# Information drawn from annual reports from the Head of the Institution’s office.

### Accountability to whom?

Respondents believed that the university structure was the platform through which one appraises faculty on social accountability activities however this appraisal is not mandatory and neither is it tracked as noted in the quote below:

"“The dean (representing the university) as well as Mulago hospital management …. do not necessarily check performance with regard to social accountability closely…am my own boss”."

Another respondent was quoted saying:

"“…achieving social accountability is more of a feeling but nothing measurable…….through the execution of my duties.”"

There were no performance indicators for social accountability; if they were, then they are not known to everyone. Indeed reporting activities of social accountability is not formally done; the indicators and the roles of individuals have not been thought through with clarity, as said by the respondent below:

"“Yes we should inform the community about our social accountability activities for their guidance, feedback and facilitation. But I think the institution on the whole does not inform the public much”."

The respondents clearly thought that personal accountability in the institution is part of social accountability. However, proper governance and compliance with administrative procedures were identified as a means to ensure quality training of graduates.

### Institutional activities supportive of social accountability

There were varied responses to the question of the strengths of the College in respect to support for social accountability. The role of teaching and research that takes place at the institution was felt to be as strength. The use of PBL and COBES as teaching methods with students who are eager to learn was thought to be a strength in teaching and learning social accountability since the students are placed in rural community sites. There is no concrete proof yet that the priority health needs of the population are being met, though it is clear that COBES has acted as an enhancing platform for the College to partner with public health facilities in health service delivery. Traditionally, integration of health service activities between the Ministry of Education and Ministry of Health has largely been nonexistent.

### Impediments to realizing social accountability

The weakness of the institution in as far as ensuring social accountability was mainly the lack of infrastructure that can sustain students in the community. There was also lack of a clear flow of information on what the community needs and how these needs would be met even though the students are capable of collecting this information while they are out in the field. Indeed the lack of a reward or punishment for institutions that are not socially accountable deters the drive forward for this important societal call.

There were gaps identified in the system that hinder implementation of social accountability in the three pillars of teaching, research and service. These challenges include lack of resources in the community to support continued students and faculty stay, resources to conduct translational research activities from a pre determined research agenda.

"“…. the gaps are big, in the implementation of the three aspects.”"

"“Most of the research being done is funder driven. But if we had money we would address priority areas.”"

It was therefore felt that there was a need to strengthen social accountability as it would produce better doctors for the community as said by a respondent:

"“…should set up objectives, sensitize staff and students…….medical issues are influenced by social issues so they should be integrated in learning and teaching activities, as a leading institution we should strengthen our position in the direction of social accountability…”"

The involvement of all hospitals in carrying out social accountability was raised and was further emphasized in the need for research conducted by postgraduate students to be oriented to community needs:

"“Social accountability should not stop at the main teaching hospital, it should be broadened…. Post graduate students may have to rotate in all hospitals surrounding the institutions”."

## Discussion

The practice of social accountability is old however the use of this term is new at MakCHS. The terms social accountability and responsiveness are used interchangeably. The scope and practice of social accountability can be expanded, by creating opportunities to ensure that medical education, research and service activities engage more community orientation [14,15].

Research in resource poor countries may be heavily influenced by funders hence creating a challenge in responding to local social needs as they may be seen in that particular environment [16].

There are weak links in the current system that retard the process of ensuring social accountability; there is a lack of feedback from the communities we serve and lack of a concerted effort by institutions to solicit for regular feedback about not only relevance of services but also for equity, quality and cost effectiveness. Indeed the lack of efficient information flow back and forth from the field undermines the mission of fully realizing social accountability.

The views expressed in this study were acts of social responsiveness however; they were not explicitly linked to meeting priority healthy needs. Quality of health care, equity issues, relevance and cost effectiveness were not mentioned. The importance of soliciting feedback was mentioned several times but, integration and partnerships with other stakeholders were omitted.

Interestingly the views expressed by the students did not differ much from those of faculty however the head of the Institution had a more detailed account on the overall big picture, the Heads of departments focused on institution programs and the students mentioned some curriculum details.

The approach we used for this survey was a rapid inexpensive form of institutional self evaluation that could be replicated by other institutions in similar contexts. It was comprehensive since it targeted key personnel with long standing experience in education but were also in administrative positions. Whereas a component of external scrutiny should have been useful, there isn’t such a mechanism per se (in Uganda) that does accreditation for social accountability or for other aspects of quality assurance regularly and in a predictable manner.

Whereas in developed countries accrediting bodies assess for social accountability regularly [17,18], the existing accreditation body in Uganda lacks the capacity for regular accreditation as may be the case in most Sub Saharan countries.

The findings of this study through conduction of an evaluation exercise albeit on a small scale fills the gap for one institution but perhaps can serve as a catalyst or as an example for others in a similar context to document through self evaluation exercises what their successes and weaknesses are in the light of social accountability.

### Limitations

Mainly heads of units and department were interviewed and their views may not fully reflect the views held by the rest of the faculty however these are individuals that run the institution and therefore can be assumed to give reliable information on what is on the ground.

The lack of a clear understanding of the definition of social accountability may have derailed the respondents. However clarification of the term was given at the beginning of the interviews.

## Conclusions

The understanding of the term social accountability was not entirely uniform however participants enumerated several facets that in turn contribute to social accountability. Whereas, there was ample evidence of the practice of social accountability, there is need to increase awareness and promote a deliberate strategy for social accountability.

## Competing interests

The authors declare no conflict of interest.

## Authors’ contributions

MG originated concept. MG and NN collected data. MG wrote first draft. NN and NKS performed critical reviews for intellectual content. All authors read and approved the final manuscript.

## Pre-publication history

The pre-publication history for this paper can be accessed here:

http://www.biomedcentral.com/1472-6920/12/96/prepub
